# A Multi‐Modal Approach to Establish Trust and Provide Value within Communities in the Deep South

**DOI:** 10.1002/alz.087583

**Published:** 2025-01-09

**Authors:** Laquetta F. Allen, Pamela Bowen, Maria L Hatch, Erik D Roberson, David S. Geldmacher, Olivio Clay

**Affiliations:** ^1^ University of Alabama, Birmingham, AL USA; ^2^ University of Alabama at Birmingham, Birmingham, AL USA; ^3^ University of Alabama, Birmingham, Birmingham, AL USA; ^4^ Alzheimer’s Disease Center, Birmingham, AL USA

## Abstract

**Background:**

Black/African Americans in the Deep South have been subjected to social segregation, discrimination, and other forms of systematic injustices that continue to negatively impact this population’s social determinants of health (SDoH). Healthy People 2030 has outlined a framework describing how adverse SDoH are associated with health inequities including higher rates of Alzheimer’s disease and related dementias (ADRD). Historically, it has been challenging to recruit citizens from this region to participate in brain aging‐related research studies.

**Method:**

Our Outreach, Recruitment and Engagement Core (ORE) utilizes a diverse team of investigators and staff to cultivate and establish trust and build a vast network of community partners. These community partners include local churches, chapters of fraternities and sororities, civic and professional organizations, library branches, business owners, and other community leaders. The ORE team has worked to create a culture of providing value in Black/African American community by using a community‐based approach to engage individuals identified by others as “hard‐to‐reach,” maintain a constant presence within these communities, provide information on access to services, and increase ADRD knowledge. This model makes it easier to build trust and get residents involved in our studies.

**Result:**

Our team demonstrates a strong commitment to actively engage communities allowing us to exceed our initial target of recruiting >40% Black/African American participants and achieve a Black/African American enrollment rate over 50%.

**Conclusion:**

Achieving greater participation by Black/African American participants in ADRD research can be accomplished through the implementation of a multi‐modal strategy that fosters community trust and instills value.

